# Separation and isolation of CD9-positive extracellular vesicles from plasma using flow cytometry†

**DOI:** 10.1039/d3na00081h

**Published:** 2023-07-13

**Authors:** Karan Khanna, Nikki Salmond, Sina Halvaei, Andrew Johnson, Karla C. Williams

**Affiliations:** a Faculty of Pharmaceutical Sciences, The University of British Columbia Vancouver V6T 1Z3 Canada karla.williams@ubc.ca; b Faculty of Medicine, UBC Flow Facility, The University of British Columbia Vancouver V6T 1Z3 Canada

## Abstract

Extracellular vesicles (EVs) are nanosized (∼30–1000 nm) lipid-enclosed particles released by a variety of cell types. EVs are found in biological fluids and are considered a promising material for disease detection and monitoring. Given their nanosized properties, EVs are difficult to isolate and study. In complex biological samples, this difficulty is amplified by other small particles and contaminating proteins making the discovery and validation of EV-based biomarkers challenging. Developing new strategies to isolate EVs from complex biological samples is of significant interest. Here, we evaluate the utility of flow cytometry to isolate particles in the nanoscale size range. Flow cytometry calibration was performed and 100 nm nanoparticles and ∼124 nm virus were used to test sorting capabilities in the nanoscale size range. Next, using blood plasma, we assessed the capabilities of flow cytometry sorting for the isolation of CD9-positive EVs. Using flow cytometry, CD9-positive EVs could be sorted from pre-enriched EV fractions and directly from plasma without the need for any EV pre-enrichment isolation strategies. These results demonstrate that flow cytometry can be employed as a method to isolate subpopulations of EVs from biological samples.

## Abstract 2

Extracellular vesicle subpopulations can be separated and isolated by flow cytometry. Isolated extracellular vesicles can be concentrated for use in downstream applications.

## Graphical abstract

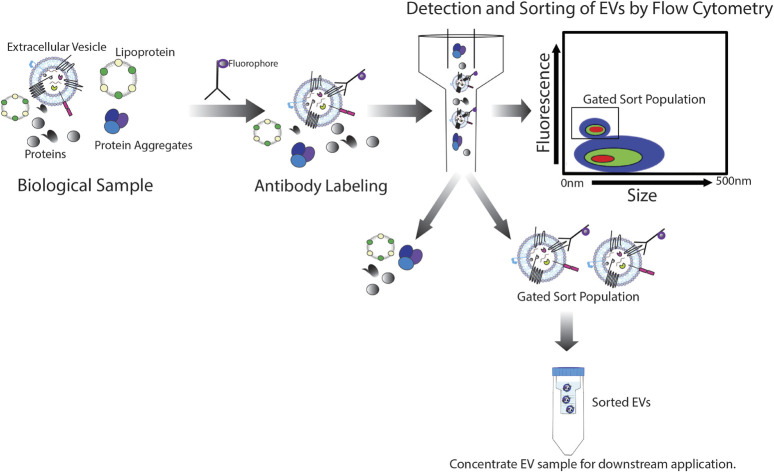

## Introduction

Developing accurate and precise biomarkers with utility for detection and monitoring of disease remains an intense area of research. Biological fluids hold an appreciable amount of information on an individual's status of health and various approaches are being pursued to detect and identify clinically relevant biomarkers. Extracellular vesicles (EVs) are nanosized lipid-enclosed particles released by all cell types into the extracellular environment and biological fluids, such as blood plasma.^1^ The diagnostic utility of EVs, in particular, arises from the detailed information they hold about their cell of origin: EVs contain lipids, proteins, and nucleic acids from the parental cell, all of which are protected from degradation by the lipid bilayer membrane.^2^ EVs, therefore, represent a source of biomarkers for disease detection, prediction of response to therapy, and prognostication.

Challenges in studying and characterizing EVs in biological fluids come from their inherent small size (∼30 to 1000 nm), making isolation and analysis difficult.^3^ Separation and isolation of EVs from biological fluids requires time-consuming procedures such as differential centrifugation.^4^ Common isolation techniques yield impure EV samples that are contaminated with other nanosized biological materials such as lipoproteins and protein aggregates. Additionally, while each EV isolation technique has unique advantages and disadvantages,^5^ they yield a heterogeneous population of EVs and analysis of specific EV subpopulations would require additional downstream processes.

Flow cytometry is a promising platform for EV isolation and has well-demonstrated utility in characterizing EVs.^6–9^ Given the multiparametric, single particle analysis capabilities of flow cytometry,^10^ this platform has the potential to support EV subpopulation isolation strategies.^11^ Accordingly, it has been shown that flow cytometry can be employed to isolate EVs from cell culture which demonstrates the feasibility of this technology for EV isolation.^11,12^ Here, we aimed to test the capabilities of flow cytometry to isolate EV subpopulations, herein CD9-positive EVs, both directly from plasma and indirectly using EVs isolated from plasma by SEC.

## Materials and methods

### Blood collection

Healthy blood samples were collected using blood collection bags by Innovative Research Inc. Four hundred and fifty milliliters of blood plus K2-EDTA were centrifuged at 5000×*g* for 15 min. The plasma was removed, aliquoted, and shipped on dry ice. Once received, samples were randomly assigned a number. All plasma samples used in this study were thawed on ice and filtered with a 0.8 μm filter to remove platelets prior to experiment. This study is compliant with all relevant ethical regulations on the use of human plasma and study approval was obtained by the institutional review board of UBC (IRB#H17-01442).

### Size-exclusion chromatography (SEC) isolation of EVs from plasma

Isolation of EVs from SEC was performed as previously described.^6,7^ Briefly, one milliliter of plasma was thawed on ice, filtered with a 0.8 μm filter, and 500 μL was run through a qEV column. The qEVoriginal/70 nm size-exclusion chromatography column (IZON Sciences Ltd) was brought to room temperature and equilibrated with two-column volumes (20 mL) of 0.2 μm filtered PBS prior to plasma application to the top of the column. On addition of the 500 μL of plasma to the column, the flow-through was collected immediately and the flow rate of the column was maintained by continuously adding 0.2 μm filtered PBS, to ensure the column was not allowed to run dry. After 3 mL flow-through was collected, a 2.5 mL EV fraction was collected and concentrated to approximately 100 μL using a 10 kDa molecular weight cut off regenerated cellulose membrane Amicon® Ultra-4 centrifugal concentrator (Millipore, Sigma).

### Nanoscale flow cytometry analysis of EVs

Nanoscale flow cytometry analysis of EVs was performed as we have previously described.^6,7^ Briefly, 2.5–10 μL of filtered plasma or isolated SEC EV-fraction were incubated with 37.5–150 ng of CF405M fluorescently labeled CD9 antibody (Abcam, ab123624), or 37.5–150 ng of CF405M mouse IgG2a antibody isotype control (Abcam, ab126036), for 30 minutes at room temperature in the dark, followed by the addition of 75–300 μL of 0.02 μm filtered PBS and transferred into a 96-well plate. Dual labeling was performed by the addition of 1 μg of PE labeled CD41 (Abcam, ab134372) or 1 μg PE mouse IgG1 isotype control (Abcam, ab91357). Samples were analyzed using the CytoFLEX S (Beckman Coulter) instrument for 60 seconds on slow (10 μL min^−1^) using 405 nm violet side scatter (VSSC) trigger. To prevent cross-contamination between samples, a PBS wash was done between each sample. The VSSC trigger threshold was set at 1027. Gain settings used: FITC – 500, BV421 – 77, and PE – 135. Isotype controls were used to guide manual gating of populations of interest.

### Flow cytometry calibration and sorting of CD9-positive EVs

A 70 μm nozzle and 60 psi was used to form a stable sorting stream on the Astrios EQ. The sample differential was set at 0.5 psi to allow for a constant flow rate of 75 000 to 95 000 events per second. The signal was triggered using the 488-FSC (forward scatter) with a 0.005% detection threshold, adjusting to allow 400 to 800 events per second as background noise. Fluorescence calibration was performed using Ultra Rainbow Quantitative Particle Kit, 6 Intensities (NIST Traceable ERF Flow Cytometry Standard) (ThermoFisher, Cat# NC1551821).^13^ For particle size estimation, 500 μL of Megamix-Plus FSC (BioCytex; Marseille, France; ref: 7802) polystyrene beads ranging from 0.1–0.9 μm were vortexed for 10 seconds and subsequently run undiluted on the Astrios EQ flow cytometer (Beckman Coulter). A GFP labelled murine leukemia virus was used as a reference biological particle.^14,15^ GFP labelled murine virus (∼124 ± 14 nm; ViroFlow Technologies) was run on the Astrios EQ in PBS and in plasma (25 μL GFP virus spiked into filtered plasma). For EV sorting, filtered plasma (100 μL) or isolated SEC EV fraction (100 μL) was incubated with 15 ng μL^−1^ of fluorescently labeled CD9 antibody, or isotype control, for 30 min at room temperature on an end-over-end rotator. Dual sorts were performed with the addition of fluorescently labeled CD41 antibody. The sample was then resuspended 1 : 30 in 0.02 μm filtered PBS and run at approximately 70 000 events per second. An enrichment sorting mode was used, as the sorting speed was too high to achieve enough EVs, due to high abort rate. The sorted populations were subsequently run on the CytoFLEX S and characterized using a CD9-CF405M and CD41-PE antibody.

### Lysis of extracellular vesicles

EVs were labelled with antibody as described in nanoscale flow cytometry analysis of EVs method section. To lyse the EVs post-labelling, 250 μL of 1% Triton-X 100 was used to dilute the sample before analysing on the CytoFLEX S.

### Concentration of flow sorted EV subpopulation

A 10 kDa molecular weight cut off regenerated cellulose membrane Amicon® Ultra-15 centrifugal concentrator (Millipore, Sigma) was used to concentrate the CD9-positive and negative sorted populations. Flow cytometry sorted EVs in ∼15 mL of PBS were added to the concentrator and centrifuged at 2000×*g* in 10 minute increments to a final volume of approximately 500 μL. If the sort volume was greater than 15 mL, 15 mL was applied to the concentrator, centrifuged to concentrate, and the remaining volume was added followed by a subsequent spin. The concentrated particles were subsequently analysed on the CytoFLEX S. To increase the yield of concentrated particles, 10 μg mL^−1^ BSA (Thermo Fisher Scientific; 23209) was added to the sorted populations prior to concentration.

### Dot blot

Protein from sorted and concentrated EVs was precipitated using trichloroacetic acid (TCA). Briefly, 125 μL of 100% (w/v) trichloroacetic acid (TCA) was added to the 500 μL concentrated EV sample and incubated at 4 °C for 10 minutes. The sample was then centrifuged at 17 984×*g* for five minutes. The supernatant was removed, leaving behind a white pellet. The pellet was washed with 200 μL of ice-cold acetone and spun in at 17 984×*g* for five minutes twice. After removing the supernatant, the pellet was dried by placing the Eppendorf tube in a 95 °C heat block for 5 minutes to remove any remaining acetone. The pellet was resuspended in 10 μL of PBS and applied onto a nitrocellulose membrane. The sample was left to dry at room temperature for approximately 20 minutes. The membrane was blocked in 5% milk dissolved in TBS-T (20 mM Tris base, 160 mM NaCl, 0.1% Tween) for 1 h and then incubated for 1 hour with primary antibodies for CD9 (BD Biosciences; 555370), CD41 (Abcam; ab83961), or ApoA1 (ThermoFisher; MA1-83002) in TBS-T at room temperature at concentrations of 1 : 500 and 1 : 1000, respectively. The membrane was washed three times with TBS-T for 10 minutes and then incubated with secondary Li-COR IRDye® 680RD and IRDye 800CW antibodies in TBS-T at a concentration of 1 : 20 000 for 1 hour at room temperature. The membrane was washed three times with TBS-T for 10 minutes and then allowed to dry before being imaged on the Sapphire Biomolecular Imager (Azure Biosystems Inc.).

### Electron microscopy

Isolated SEC EV fraction and isolated EVs from flow cytometry were fixed in 2% electron microscope grade paraformaldehyde (Fisher Scientific) in PBS and adsorbed onto formvar/carbon-coated 200 mesh nickel grids for approximately 1 minute. Grids were negatively stained by incubation with pre-filtered 1% uranyl acetate (Fisher Scientific) pH 4.6 for 30 seconds. Grids were blotted dry before being imaged using a Helios NanoLab 650, fitted with a STEM detector (Thermofisher, Systems for Research, Kanata, ON, Canada) in scanning transmission bright field imaging mode at 30 kV.

### Nanoparticle tracking analysis

The size and concentration of collected particles were analysed by nanoparticle tracking analysis (NTA) using a NanoSight LM10 equipped with a blue 488 laser and sCMOS camera (Malvern Panalytical). The data were acquired and analysed using 3.3.301 software. Samples were diluted 1 : 10–1 : 100 in PBS and injected into the NTA analysis chamber. A syringe pump at speed 40 units was used to create a continuous flow of sample through the camber during data acquisition of three 30 second videos at camera level 14. Particles were tracked and analysed using detection threshold 3.

### Light scatter calibration

The light scatter calibration for each flow cytometer was performed using Rosetta Calibration beads as per manufactures guidelines (Cal003, Exometry, The Netherlands). Mie modeling, conversion of light scatter to diameter was performed using Rosetta Calibration software (v2.03) as per manufacturer's guidelines. This allowed for the conversion of light scatter intensities from arbitrary units to nanometers using Mie theory core/shell model. For this, certain assumptions were made regarding the refractive index of EVs and the core refractive index was set at 1.38, the shell refractive index at 1.48, and the particle shell thickness at 6 nm.^16^ Size calibration performed on the murine leukemia virus assumed a refractive index of 1.51.^17^ After calibration, the files were imported and analyzed using FlowJo (v10.8.1).

### Fluorescent calibration

Ultra Rainbow Quantitative Particle Kit, 6 Intensities (NIST Traceable ERF Flow Cytometry Standard, ThermoFisher, Cat# NC1551821) calibration beads were used to calibrate the fluorescent channels of both the CytoFLEX and Astrios EQ using a linear regression of the log-transformed mean fluorescent intensity and the known equivalent reference fluorophores: fluorescein, Nile Red, and Coumarin 30. The data was then converted using the derived slope and intercept using FlowJo (v10.8.1) derived parameter function.

### Software, statistical analysis and data acquisition

Nanoscale flow cytometry data and images were acquired using the CytoFLEX CytExpert 2.3 and FlowJo (v10.8.1) software. Figures were prepared with Adobe Illustrator 24.1.2. Data was handled in Excel and analyzed using GraphPad Prism 8.0.1. AzureSpot Pro was used to visualize the dot plots imaged on the Azure Sapphire Biomolecular Imager. Distribution of collected data was analyzed for normality using Shapiro–Wilk. Data that was proven to normally distribute was analyzed using parametric Student's *t*-test, or paired *T*-test.

## Results

### Analysis and isolation of nanosized particles by flow cytometry

**Fig. 1:**
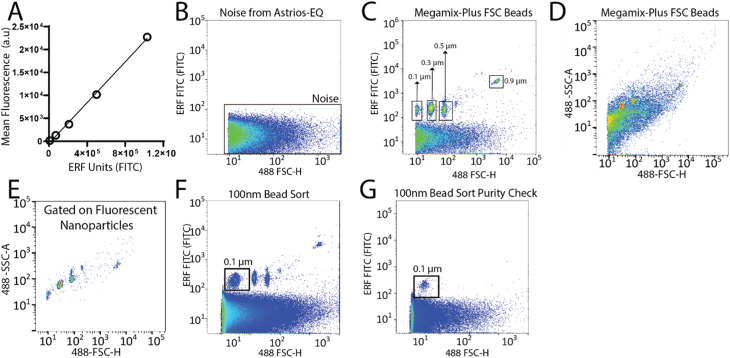
Detection and sorting of nanoparticles using the Astrios EQ flow cytometer. (A) Calibration plot using SPHERO™ Rainbow Calibration Particles with known Equivalent Number of Reference Fluorophores (ERF) value for each bead plotted against their fluorescence intensity (arbitrary units). (B) Representative image of the baseline noise detected from the Astrios EQ when running deionized water in the fluorescent FITC channel. (C and D) Megamix-Plus FSC polystyrene beads ranging from 0.1, 0.3, 0.5, and 0.9 μm triggered using 488 FSC-H and analyzed on: (C) the FITC channel and (D) 488-side scatter (488-SSC-A). (E) Gated fluorescent beads analyzed by 488-SSC-A. (F) Selection of 0.1 μm nanoparticles from the Megamix-Plus FSC beads for sorting using the Astrios EQ. (G) Sorted 0.1 μm beads analyzed by the Astrios EQ to confirm isolation of the 0.1 μm nanoparticles from the Megamix.

To set-up the Astrios EQ flow cytometer for the detection and sorting of small particles we analysed the background noise of the machine, performed bead calibration, and assessed the detection of a biological reference material: ∼124 nm GFP-labelled virus.^15^ Our study was performed with reference to MIFlowCyt-EV recommendations.^13^ FITC fluorescence calibration was completed using NIST Traceable ERF (Equivalent Number of Reference Fluorophores) Flow Cytometry beads (Fig. 1A). Deionized water was run through the system while triggering on the 488-FSC channel to determine baseline noise, which showed a low level of background noise from the instrument (Fig. 1B). Following this, the instrument was calibrated using Megamix-Plus FSC beads (BioCytex) with sizes ranging between 0.1, 0.3, 0.5, and 0.9 μm to ensure different sized fluorescent nanoparticles could be efficiently resolved. Megamix-Plus FSC beads have demonstrated use in flow cytometry calibration for small particle analysis.^18^ From this, it was determined that at a sample differential pressure of 0.5 psi and a 488-FSC trigger of 0.005% threshold, resolved these fluorescent nanoparticles (Fig. 1C). FSC was found to have improved particle resolution over SSC and was selected as the trigger channel (ESI Fig. 1A and B†). Nanoparticles were readily detected by fluorescence (Fig. 1C) but difficult to resolve by scatter alone (Fig. 1D). Gating on the fluorescent nanoparticles was required to resolve the particles from background noise (Fig. 1E). The 0.1 μm fluorescent nanoparticle population was gated (Fig. 1F) and sorted. Sorted 0.1 μm fluorescent nanoparticles were analyzed for purity and enrichment (Fig. 1G) as an initial proof of concept.

**Fig. 2:**
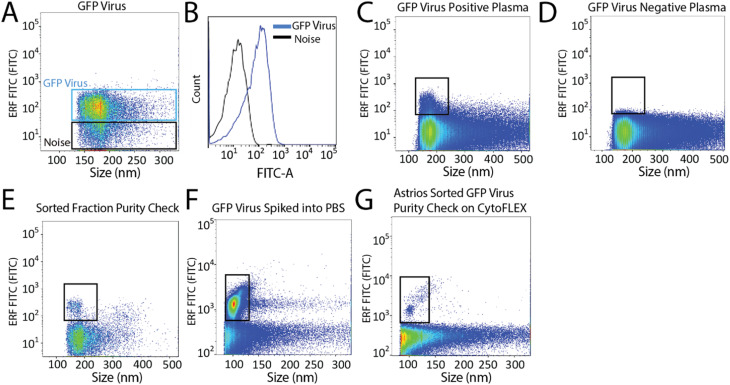
Detection and sorting of GFP-viral particles using the Astrios EQ flow cytometer. (A) GFP virus detection using the FITC-A channel. (B) Overlay plot of the GFP virus and the background noise from the Astrios EQ. (C) 25 μL of GFP virus spiked into 100 μL of filtered plasma analyzed on the Astrios EQ. (D) Plasma, without GFP virus spike, analyzed using the fluorescent FITC-A channel. (E) Sorted GFP virus analyzed using the Astrios EQ flow cytometer. (F) GFP virus diluted in PBS and analyzed on a nanoscale flow cytometer analyzer (CytoFLEX S). (G) Sorted GFP virus analyzed by a nanoscale flow cytometer analyzer (CytoFLEX S).

Next, we aimed to sort a biological reference material. GFP labelled murine virus was used as a biological reference material and analyzed on the Astrios EQ (Fig. 2A and B). Scatter calibration was performed to enhance data reporting and interpretation,^19,20^ and as per MIFlowCyt-EV recommendations.^13^ Light scatter calibration was performed for the Astrios EQ and CytoFLEX S using the scatter-diameter relationship, which is dependent on a particles refractive index, to convert arbitrary units into nanometers (ESI Fig. 2A and B†).^21^ To determine if a biological reference material could be sorted from plasma, GFP-viral particles were spiked into 0.8 μm filtered plasma and analyzed by Astrios EQ flow cytometry. GFP-viral particles were found to be readily detected above background (Fig. 2C) when compared to GFP-virus negative plasma (Fig. 2D). Next, we aimed to sort the GFP viral particles from plasma. GFP-virus spiked into plasma was gating on (as shown in Fig. 2C) and the positive population was sorted. The sorted population was assessed by flow cytometry to confirm successful sorting of virus (Fig. 2E). A nanoscale flow cytometry analyzer, optimized for small particle detection (CytoFLEX S instrument triggered on VSSC), was also used to validate the sorting of the GFP virus. Nanoscale flow cytometry readily detected GFP viral particles diluted in PBS (Fig. 2F) and further confirmed the successful sorting of the GFP virus (Fig. 2G).

### Analysis and isolation of CD9-positive extracellular vesicles by flow cytometry

**Fig. 3:**
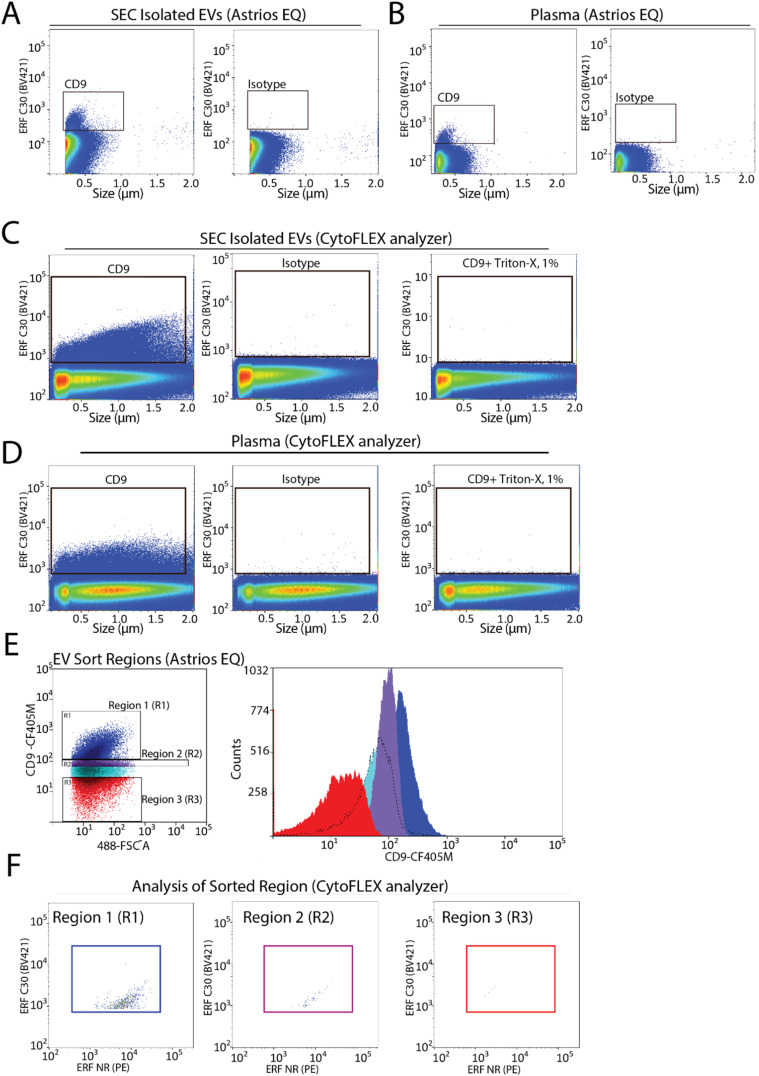
Identification and isolation of CD9-positive EVs from plasma using flow cytometry. (A) Representative image of CD9-positive population from isolated EVs on the Astrios EQ and its respective isotype control. *n* = 2. (B) Representative image of CD9-positive population from filtered plasma on the Astrios EQ and its respective isotype control. *n* = 2. (C) Representative image of CD9-positive population from isolated EVs on the CytoFLEX S, its isotype control, and lysed with 1% Triton-X 100. *n* = 2. (D) Representative image of CD9-positive population from filtered plasma on the CytoFLEX S, its isotype control, and lysed with 1% Triton-X 100. *n* = 2. (E) CD9 immunostained plasma gated into three different regions followed by sorting of each region. (F) Sorted regions of interest (R1, R2, R3) analysed and characterized on the CytoFLEX S by CD9 and CD41 immunostaining.

To assess the capabilities of flow cytometry to sort EV subpopulations from plasma we focused on CD9-positive EVs, as CD9-positive EVs are known to be abundant in plasma.^7,22^ Fluorescence calibration was performed for phycoerythrin (PE) and Pacific Blue (PB) using NIST beads with assigned ERF values (ESI Fig. 2C–F†). EVs were isolated by size-exclusion chromatography (SEC) from 0.8 μm filter plasma and analyzed for CD9-positive EVs by flow cytometry. A CD9-positive EV population was readily detected by flow cytometry (Fig. 3A). Next, detection of CD9-positive EVs using 0.8 μm filtered plasma without pre-enrichment/isolation was assessed and a CD9-positive population was detected (Fig. 3B). Absence of the swarm phenomenon was tested using a plasma dilution series (ESI Fig. 3A†). The number of events detected decreased relative to the plasma dilution factor suggesting that single EV events are detected. CD9-positive EV populations were further validated using the CytoFLEX S nanoscale flow cytometry analyzer. Using 0.8 μm filtered plasma, CD9-positive EVs were analysed directly from plasma and from pre-enriched SEC isolated EVs (Fig. 3C and D). CD9-positive EVs were readily detected in SEC isolated EV samples and in plasma samples. As EVs are lipid bilayer enclosed particles, they can be distinguished from protein aggregates by their ability to lyse when treated with different detergents. EVs treated with 1% triton-X should lyse and no longer be detected by nanoscale flow cytometry while protein aggregates, on the other hand, would still be detectable. Therefore, detergent lysis of EVs is a common technique used to provide evidence that particles being detected and analysed are EVs.^23^ This is also a recommendation of MIFlowCyt-EV.^13^ The addition of detergent treatment to samples resulted in a complete loss of CD9-positive events strongly suggesting that CD9-positive events detected by flow cytometry are EVs (Fig. 3C and D). For all conditions, an isotype control for CD9 was used to ascertain CD9 antibody specificity and used to manually set gates on the EV population of interest.

To determine the sort region for CD9-positive EVs, we separated the CD9-immunostained plasma sample into three regions (Fig. 3E). Each region was sorted and the sorted particles were analyzed and characterized by nanoscale flow cytometry. As we previously reported a significant fraction of CD9-positive EVs in plasma are CD41-positive, which suggests they are of platelet origin, we analysed each sort region for CD9 and CD41 (Fig. 3F).^7^ Region 1 (R1) was found to contain the majority of CD9-positive EVs, which were also positive for CD41. Only a few events were identified in R2 and R3. Overall, this identified R1 as the region to sort CD9-positive EVs.

**Fig. 4:**
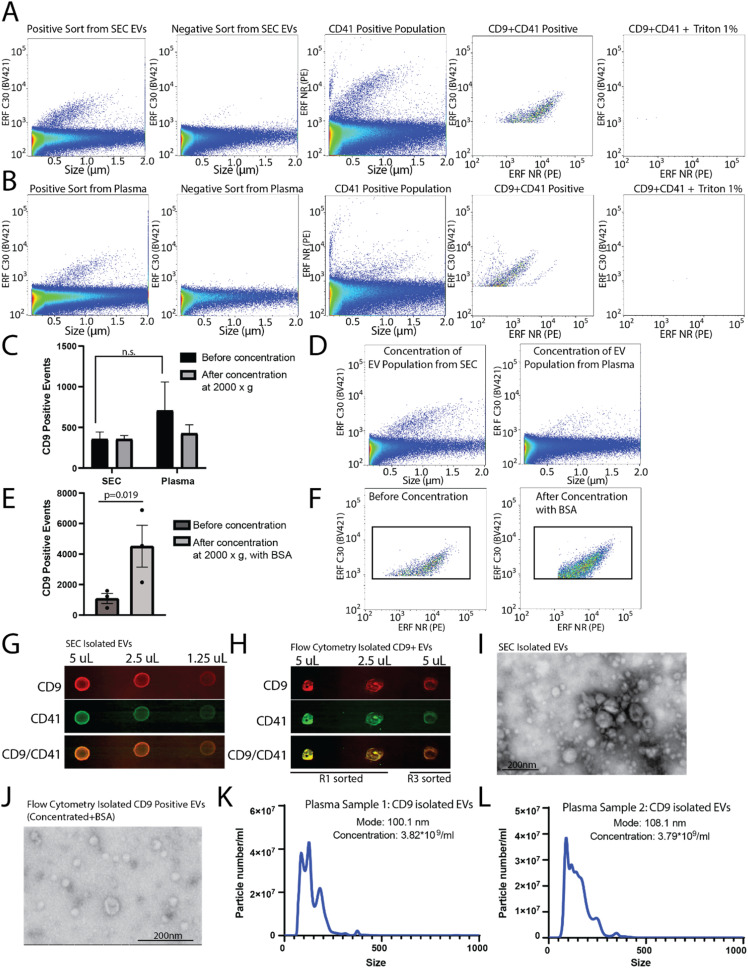
Analysis and characterization of CD9 sorted populations. Flow cytometer analyser (CytoFLEX S) analysis of sorted EVs from: (A) SEC isolated EV fractions from plasma and (B) 0.8 μm filtered plasma. (A and B) CD9-positive and negative sorts analysed by CD9 immunostaining. EV characterisation performed by CD9 and CD41 dual immunostaining. EV lysis by 1% Triton-X 100. *n* = 2. (C) Quantification of CD9-positive EV events, by nanoscale flow cytometry, from flow cytometry sorts using before and after concentration. *n* = 2. (D) Representative flow plots of CD9-positive EV populations post-concentration. (E) Quantification of CD9-positive EV events from flow cytometry plasma sort before and after concentration with 10 μg mL^−1^ BSA. *n* = 3. (F) Representative plots of sorted CD9-positive EVs from plasma before concentration and post-concentration with 10 μg mL^−1^ BSA. *n* = 2. (G and H) Dot blot for CD9 (*n* = 2) and CD41 using (G) SEC isolated EVs and (H) flow cytometry isolated CD9-positive EVs post-concentration with 10 μg mL^−1^ BSA. (I and J) Scanning transmission electron microscopy images of EVs isolated by (I) SEC and (J) EVs isolated by flow cytometry sort of CD9-positive events, post-concentration with 10 μg mL^−1^ BSA. (K and L) Nanoparticle tracking analysis on sorted CD9-positive EVs from plasma post-concentration plus 10 μg mL^−1^ BSA from two different plasma samples.

To determine the capacity to sort EVs from a pre-isolated SEC EV fraction *versus* plasma, plasma samples were filtered and divided into two aliquots, one of which was used for SEC EV isolation. SEC isolated EVs and plasma samples were immunostained for CD9, and sorted for R1 (positive population) and R3 (negative population) (as shown in Fig. 3E and F). A CD9-positive population was readily identified from the SEC CD9-EV sort (Fig. 4A) and the plasma CD9-EV sort (Fig. 4B). CD9-positive EVs were also characterized as CD41-positive demonstrating the ability to sort and isolate a specific EV population which can be further characterized using additional markers (Fig. 4A and B). EVs were validated through detergent treatment to lyse EV lipid bilayers. We also assessed the capability of flow cytometry to perform multiparametric sorting. Plasma samples were labeled with CD41 and CD9 antibodies and sorted for dual positive events (ESI Fig. 3B and C†). From a single EV marker sort (*i.e.* CD9-positive sort) the percentage of isolated EVs found to be positive for CD41 was approximately 78%. Sorting for dual positive EVs (CD9-CD41 positive events) resulted in approximately 98% of the sorted events being CD9 and CD41 dual positive.

These results demonstrate that flow cytometry can be optimized for sorting EV subpopulations of interest. However, the average run can take up to 3–8 hours and the final EV collection is present in ∼15–20 mL of buffer. To perform downstream analysis on EV subpopulations, concentration of the sample is required. Amicon® Ultra-15 centrifugal concentrator with a 10 kDa molecular weight cut off were used to concentrate the large volume (∼15 mL) of sorted EVs to enable downstream analysis. Initial concentration attempts spinning at 4000×*g* resulted in complete loss of the sample (data not shown). Reducing the spin speed to 2000×*g* facilitated volume reduction and sample retention; however, no enrichment of EVs in the concentrated samples was found (Fig. 4C and D). Recently it was demonstrated that high purity EV preparations in PBS may be prone to aggregation and to absorption onto surfaces.^24,25^ As such, to improve yield during concentration 10 μg mL^−1^ BSA was added to each sample prior to centrifugation and concentration. Using this method, there was significant enrichment of CD9-positive EVs post-concentration (Fig. 4E and F). The addition of 10 μg mL^−1^ BSA to the sample did not change how the CD9 positive population appeared on the nanoscale flow cytometer (data not shown).

CD9-positive flow cytometry sorted samples were concentrated to ∼500 μL and the protein was precipitated out of the sample using trichloroacetic acid and acetone. As a positive control, EVs from plasma were also collected using SEC, concentrated, and the proteins precipitated. The precipitated protein from the SEC concentrated EVs and the flow cytometry isolated CD9-positive EVs were resuspended in 10 μL and dropped onto a nitrocellulose membrane at 50% decreasing concentrations in equivalent volumes (Fig. 4G and H). In addition to the positive sort population (R1), the negative sort population (R3) was also subjected to the same concentration and precipitation protocol (Fig. 4H). The membrane was probed for CD9 and CD41, which was found in the EVs collected using SEC from plasma, and was enriched in the CD9 sorted samples (R1) as compared to the negative sort population (R3) (Fig. 4H). EV-preparation contaminating protein, Apolipoprotein A1, was also analysed in CD9-positive sorted EVs and SEC isolated EVs by dot-blot (ESI Fig. 3D†). EVs isolated by SEC and flow cytometry were analysed using scanning transmission electron microscope (STEM) (Fig. 4I and J). To assess the size range of the CD9-positive sorted EVs, NTA was performed on CD9-sorted EVs from two different donors (Fig. 4K and L). The mode size for CD9-positive EVs was found to be ∼100 nm. Overall, this demonstrates that CD9-positive EVs can be isolated using flow cytometry and concentrated for use in downstream analysis platforms.

## Discussion

The use of EVs as a platform for disease detection and monitoring holds great potential. EVs are stable and readily detectable (2–6 × 10^10^ EVs per mL in plasma), supporting their utility in clinical applications.^2^ Plasma-derived EVs can be stored at −80 °C with long-term stability and can undergo multiple freeze–thaw cycles without negative impacts on EV number or biomarker loss.^26^ These are significant advantages for biomarker-based assessment of clinical samples. All components of the EVs (proteins, nucleic acids, lipids, glycans) can be studied retrospectively from large cohorts of stored patient plasma. The aforementioned suggests that an EV-based liquid biopsy may be advantageous over other approaches, such as circulating tumor cells and cell free nucleic acid analysis.^27–31^ EVs are therefore an important and accessible source of physiological information regarding health and disease.

Biological fluids such as plasma contain EVs from diverse cell types, therefore, isolation of cell specific EV populations from biological fluids remains challenging.^32^ Methods to date have often involved affinity-based strategies.^33,34^ Affinity-based techniques often employ immunocapture platforms to isolate a specific population of EVs.^35^ The advantage of using immunocapture based approaches is the ability to isolate biomarker-specific EVs, which may further aid in identifying disease-specific biomarkers. Although currently useful for cell culture-derived EVs, this technique can have low yields from biological samples such as plasma depending on the immunocapture target.^36–38^ One of the most commonly used immunoaffinity based approach utilizes antibodies coupled to magnetic beads, often targeting surface tetraspanins enriched on EVS such as CD9, CD63, CD81.^38–41^ However, utilization of bead-based systems requires challenging elution that may disrupt EV integrity.^38,42^ As such, this system cannot address questions related to the biological function of sorted or isolated EV sub-populations. Employing bead-based immunocapture techniques in complex biofluids, such as plasma, would also likely be challenging due to non-specific bead interactions and low capture efficiency.

Other EV isolation techniques have also been utilized, but often require unique or lab-generated equipment such as asymmetric flow field-flow fractionation to isolate small particle populations based on size (exomeres and exosomes),^43^ immunomagnetic sequential ultrafiltration (iSUF) for isolating subpopulations of EVs,^44^ and microfluidic devices,^45^ which, while useful, may not necessarily be suitable for further downstream analysis. Common EV isolation techniques such as SEC, differential centrifugation, commercially available kits, each have limitations,^5^ and it has been reported that different EV preparation methods can result in distinct size distributions in the isolated population.^46^ This suggests that different methods may result in distinct populations being used for downstream analysis platforms.

Isolation of EVs by flow cytometry is potentially an excellent platform for bulk EV isolation and EV subpopulation isolation; however, the resolution of conventional flow cytometers has generally been considered too low for the detection of EVs.^47^ This has resulted in researchers using dedicated flow cytometers optimized for small particle analysis. However, newer generation flow cytometers challenge this with improvements in particle resolution. This is an advantage as many research centres have common flow cores, and where newer generation flow cytometers are in place, these centres would have the ability to optimize flow cytometry for EV isolation. This would be a useful technique that could be employed by these core facilities and, thus, enable any researcher to perform bulk or subpopulation EV isolation strategies. This could have significant advantages over other EV isolation strategies. For example, ultracentrifugation used to isolate EVs may alter EV functionality;^48^ thus, hindering functional studies. Flow cytometry, however, would likely enable the sorting of total EVs from cell culture or biological fluids, *via* dyes such as CFSE, while maintaining the structure and function of individual EVs.^11,12^ Flow cytometry isolation of EV subpopulations for downstream functional studies could be hampered by the presence of a fluorescently-labelled antibody, however bound antibodies would not affect EV proteomic, glycomic, or genomic analysis. This would enable a more in-depth analysis of EV content in relation to the isolated subpopulation. Potentially, this strategy could be used to improve on the characterization exosomes and ectosomes and to support small scale biomarker discovery studies for diagnosis, prognostication, and monitoring of disease.

Here we report the optimization of flow cytometry for the isolation of EV subpopulations. Immunolabeled CD9-positive EVs were sorted using SEC isolated EVs and using plasma, without the need for any pre-enrichment strategies, by flow cytometry. Optimization of sample concentration post-sort resulted in small volumes of a enriched EV subpopulation with utility for downstream applications. Interestingly, analysis of CD9-positive EVs by NTA found the mode size to be ∼100 nm and EM images also suggest a small size range for CD9-positive EVs. Potentially, this is just a result of the small number of samples we used for our sorting and analysis; analysis of a larger cohort could demonstrate a higher degree of size variability between individual plasma samples. The size of the isolated CD9-positive EV could also be a result of their biogenesis. CD9 is considered a marker of small ectosomes and EV isolation by ultracentrifugation has demonstrated that CD9 is abundant in 200k pellet (small ectosomes and exosomes) with low levels found the 10k pellet (large ectosomes and oncosomes).^49^

As we previously identified CD9-positive EVs as a relatively abundant in plasma,^7^ we initially focused on sorting this population. Altered CD9 expression in tumor cells has been shown in multiple cancer types with contradictory evidence on the role of tumor cell associated CD9 in cancer progression.^50–52^ Expression on EVs, however, appears to support a role for CD9-positive EVs in cancer detection and progression. In plasma, CD9 EV levels have been shown to be elevated in prostate cancer patient plasma samples relative to healthy controls.^53^ Expression analysis using multiple breast and prostate cancer cell lines found CD9 to be enriched in all cell line EV preparations.^54^ Interestingly, while CD9 was abundant in all EV preparations, the cell line expression of CD9 varied. In the tumor microenvironment, other cell types may contribute to total CD9 EV levels. In pancreatic ductal adenocarcinoma (PDAC), stromal expression of CD9 was associated with poor patient outcomes and cancer associated fibroblasts were found to release CD9-positive EVs which induced PDAC progression.^55^ In addition to CD9 EVs derived from tumor and stromal cells, there is evidence to support tumor cell activation of platelets and increased platelet EV release.^56,57^ Our current, and previous, work demonstrates that over half of the CD9 EVs in plasma are of platelet origin leaving the remaining being derived from other cell sources. As our study demonstrates the capability of flow cytometry to isolate CD9 subpopulations, this technology could be used for future studies to isolate and examine either platelet-derived EVs or CD9 non-platelet-derived EVs in individuals with a status of health *versus* disease. Additionally, other EV subpopulations using markers for tumor-derived, immune-derived, or other EV subpopulations could be of interest.

There are some limitations to our study. While our study was performed using triggering on 488-FSC other studies have suggested that triggering on 488-SSC provides better resolution of small particles.^11^ SSC is generally considered to have a higher sensitivity for small particle detection and resolution and we routinely use VSSC for our studies using a nanoscale flow cytometer analyser.^6,7^ In this study, and in-regards to the flow cytometer sorter, triggering on 488-FSC showed improved resolution over 488-SCC which directed us to perform sorts using FSC. However, we did not perform test sorts for EV isolations using 488-SSC, or VSSC, to compare EV-sorting capabilities, efficiency, and composition of SCC *versus* FSC sorted populations. Another potential limitation of our study is the time per sort as we found that flow cytometry sort times ranged from 3 to 8h. Samples with lower event rates would likely permit a faster sort time and we did note a faster sort time for SEC isolated EVs compared to plasma. Removal of excess, unbound, antibody could also result in a cleaner sample and reduce the total sort time. A protocol for this has been recently described where the use of an EV-Clean method was capable of removing unbound labels and this improved flow cytometry EV analysis.^58^ Future studies should be aimed at addressing these limitations and include additional analysis, such as proteomics, to assess EV-subpopulations isolated by flow cytometry.

Overall, this work demonstrates that an EV subpopulation can be isolated and enriched from a biological sample. Importantly, our isolation strategy could be implemented by any flow core facility using the methods we described. While we performed purity checks on a dedicated nanoscale flow cytometer, as sorting in the small particle range becomes more common, validation of EV sorts could be performed through more standard EV checks (such as western blot and EM). Given that EVs contain detailed information about their cell of origin, the isolation of specific EV-subpopulations could support downstream omics analysis platforms. Interrogating the content of unique tissue-specific EV subpopulations may yield novel biomarkers with clinical utility to improve methods of disease detection, monitoring, or prognostication.

## Ethics declaration

Ethics approval and consent to participate.

## Ethics approval

Study is compliant with all relevant ethical regulations on the use of human plasma. Study approval was obtained by the institutional review board of UBC (IRB#H17-01442).

## Consent for publication

All the authors agree to the content of the paper and are being listed as a co-author of the paper.

## Data availability

All data supporting the conclusions of this research is provided in this article and in supplemental files. All flow files associated with this work can be found at FlowRepository.og, study IDFR-FCM-Z5GG. Link: https://flowrepository.org/id/RvFrwzbaMuNMxHcoTdqJS0fNGDcAU8MscWZWpnXJuGOL5cCYNmDTRgjJMjftRWz9. Any other raw data in this current study is available from the corresponding author on reasonable request.

## Author contributions

KK, SH, and NS conducted experiments. KK and AJ optimized the flow cytometry sort in the small particle size range. KK, KCW, and NS analyzed the data. KCW and KK conceptualized the manuscript and KCW provided direction and guidance in assembling the manuscript. KK drafted the manuscript and KCW, NS, and KK revised the manuscript. All authors reviewed and approved the manuscript.

## Conflicts of interest

The authors declare no potential conflicts of interest.

## Supplementary Material

NA-005-D3NA00081H-s001
