# Supplementary material for: Separation and isolation of CD9-positive extracellular vesicles from plasma using flow cytometry
Source: Nanoscale Adv. 2023 Jul 13;5(17):4435–46. doi: 10.1039/d3na00081h (PMC10448347; doi:10.1039/d3na00081h)
Supplement: NA-005-D3NA00081H-s001 [file NA-005-D3NA00081H-s001.pdf]

Electronic Supplementary Material (ESI) for Nanoscale Advances.
This journal is © The Royal Society of Chemistry 2023

SUPPLEMENTAL FIGURES

Separation and Isolation of CD9-Positive Extracellular Vesicles from Plasma Using Flow Cytometry

Karan Khanna,[1] Nikki Salmond,[1] Sina Halvaei,[1] Andrew Johnson,[2] and Karla C. Williams[1,4]

[1]Faculty of Pharmaceutical Sciences, The University of British Columbia, Vancouver, V6T 1Z3, Canada

[2]Faculty of Medicine, UBC Flow Facility, The University of British Columbia, Vancouver, V6T 1Z3, Canada

[4]Correspondence to: karla.williams@ubc.ca

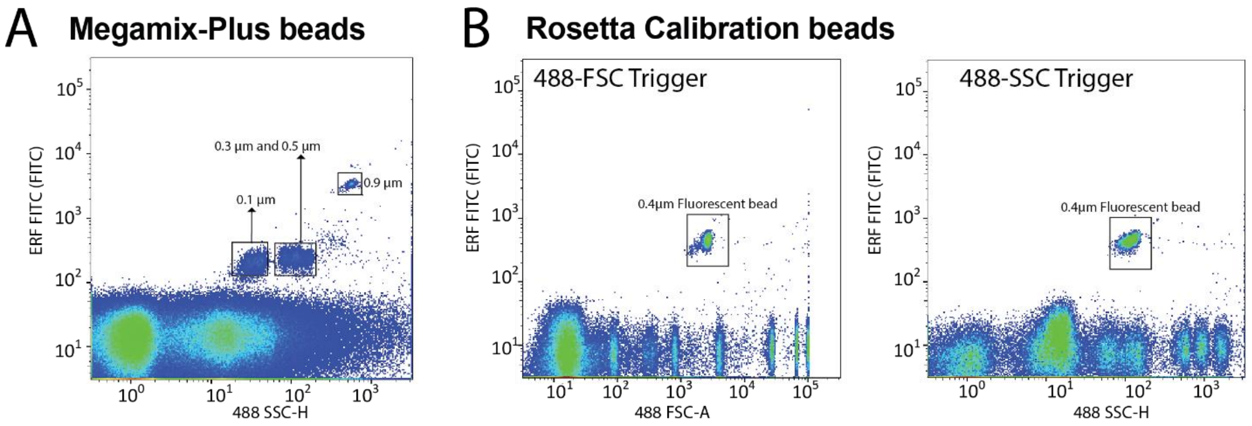

Figure S1: **(A)** Megamix-Plus polystyrene beads triggered using 488 SSC-H analyzed on the FITC channel. **(B)** Rosetta calibration beads analysed on the Astrios EQ using different triggering strategies. Left plot, resolution of beads when triggered using 488-FSC. Right plot, resolution of beads when triggered using 488-SSC.

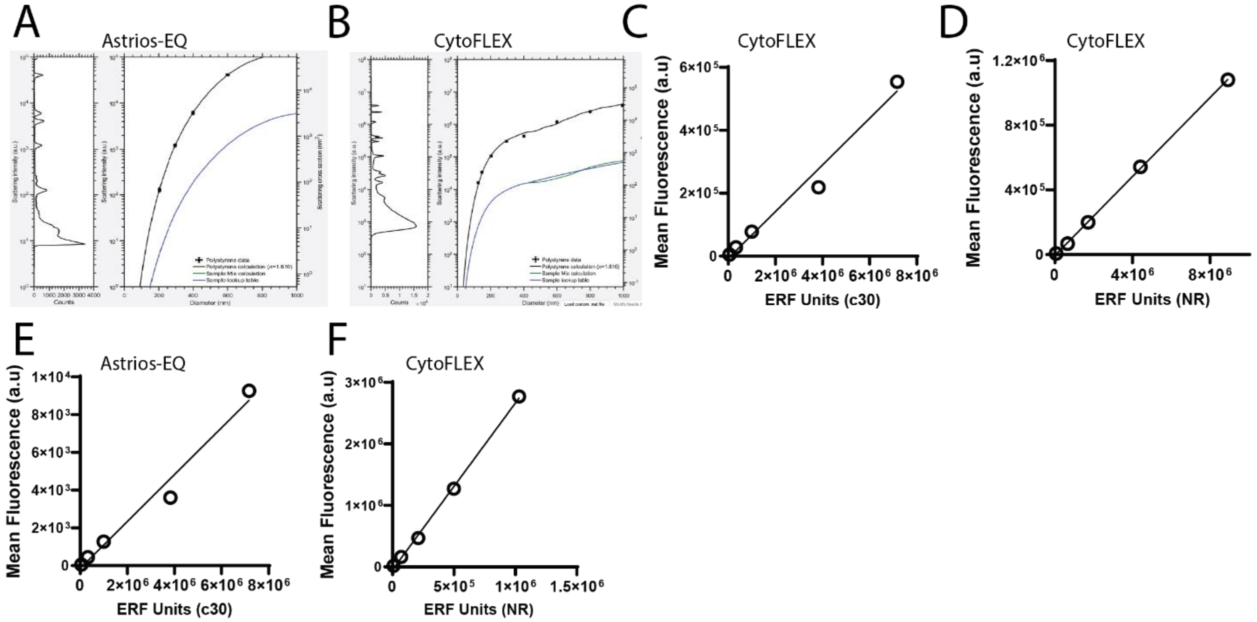

Figure S2: Calibration plots for the Astrios-EQ **(A)** and CytoFLEX **(B)** using Rosetta beads and calibration software. **(C-F)** Calibration plot using SPHERO™ Rainbow Calibration Particles with known Equivalent Number of Reference Fluorophores (ERF) value for each bead plotted against their fluorescence intensity (arbitrary units).

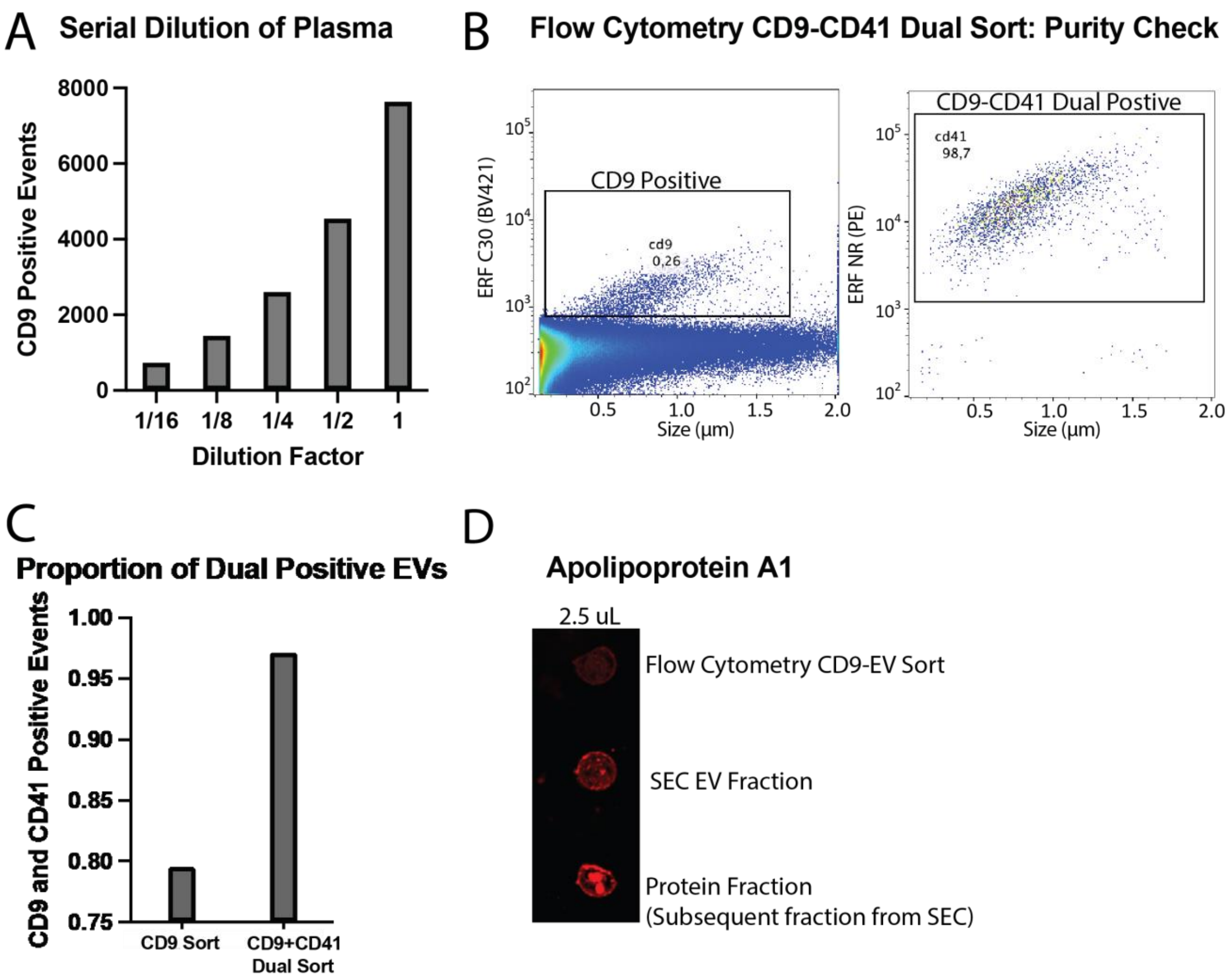

Figure S3: CD9 extracellular vesicles sorting by flow cytometry. **(A)** The presence of swarming as assessed by plasma serial dilution and quantification of events by flow cytometry. **(B)** Flow cytometry CD9-C41 dual positive EVs run on a flow cytometry analyzer. Left plot shows CD9-postive events and right plot shows the number of those events that are dual positive by CD41 immunostaining. **(C)** The proportion of dual positive CD9-CD41 EVs from a CD9-positive sort and a CD9-CD41 dual positive sort (n=2). **(D)** The presence of a common protein contaminant, Apolipoprotein A1, probed on a dot blot (n=2) using CD9-postive flow cytometry isolated EVs (top row), SEC isolated EVs (middle row), and the SEC proteinaceous fraction, collected after the EV fraction, (bottom).
